# Fabrication of Ordered Mesoporous Silica/Polyethersulfone Mixed-Matrix Membranes for Improved Removal of Middle-Molecule Toxins Within Hemodialysis

**DOI:** 10.3390/membranes16070250

**Published:** 2026-07-21

**Authors:** Rongrong Ji, Peiyan Shi, Ting Dong, Wenjie Hou, Kangjian Tang

**Affiliations:** 1 Innovation Center for Chemical Science, College of Chemistry, Chemical Engineering and Materials Science, Soochow University, Suzhou 215123, China; jirongronghb@163.com (R.J.); 9855428047@163.com (P.S.); 2 Department of Nephrology, The Fourth Affiliated Hospital of Soochow University, No. 9 Chongwen Road, Suzhou 215123, China; dongting@suda.edu.cn (T.D.); wjhou@suda.edu.cn (W.H.)

**Keywords:** polyethersulfone, mixed-matrix membrane, hemodialysis, middle-molecule toxin reduction

## Abstract

As the core component of an artificial kidney, a hemodialysis membrane can remove metabolic wastes and excess fluid from the blood while retaining essential proteins. Despite their essential role in blood purification, current hemodialysis membranes still show limited efficiency in clearing middle-molecule uremic toxins, especially β_2_-microglobulin. Ordered mesoporous silica (SBA-15) was used as an inorganic pore-regulating additive to construct ordered mesoporous silica/polyethersulfone (PES) mixed-matrix membranes for separation applications. The incorporation of SBA-15 may help form additional effective transport pathways in the PES membrane by regulating pore formation, increasing membrane hydrophilicity, and improving apparent pore connectivity, thereby reducing the apparent transport resistance of middle-molecule solutes. As a result, the composite membranes achieved improved dialysis performance while maintaining high BSA retention. The SBA-15 loading was systematically optimized. Relative to the pristine PES membrane, the 7 wt.% SBA-15 membrane reduced the water contact angle from 65.1° to 49.0° and increased lysozyme reduction from 40.9% to 55.2%, with pure water permeability reaching 261.5 L m^−2^ h^−1^ bar^−1^ and bovine serum albumin (BSA) retention remaining above 90%. These results suggest that SBA-15 may regulate the pore structure of PES membranes and improve apparent pore connectivity, thereby facilitating middle-molecule solute transport while maintaining high BSA retention.

## 1. Introduction

Hemodialysis remains one of the principal modalities of kidney replacement therapy for patients with end-stage kidney disease [1,2,3,4,5]. The dialysis membrane is the central component of a hemodialysis system and plays a key role in solute removal and protein retention [6,7,8]. Uremic toxins are generally classified according to molecular weight as small water-soluble solutes or middle molecules. The former, including urea and creatinine, usually have molecular weights of less than 2000 Da, whereas the latter, represented by β_2_-microglobulin, typically fall within the range of 2000−60,000 Da [9]. As a large plasma protein of approximately 66 kDa, serum albumin should be retained during membrane separation. Although current dialysis membranes are generally effective in removing small solutes, their ability to remove middle-molecule toxins remains insufficient. This limitation has become an important challenge in further improving dialysis adequacy [10,11,12].

This problem is not simply a matter of membrane flux. For middle molecules, transport is strongly affected by the continuity, accessibility, and tortuosity of the pore network. Poorly connected or constricted pores can reduce the number of usable transport pathways and lengthen the diffusion route, leading to relatively high transmembrane mass-transfer resistance [12]. At the same time, widening the sieving window may increase albumin loss. Therefore, high-performance membranes should combine a suitable effective pore-size distribution with good pore connectivity and low pathway tortuosity so that middle-molecule transport can be improved without reducing protein retention.

Most earlier PES- or PSF-based membranes were improved through phase-inversion control and the use of hydrophilic modifiers such as PVP and PEG, with the aim of facilitating middle-molecule transport [13,14]. Su et al. [15] reported that a PES/PVP dialysis membrane reduced β_2_-microglobulin by about 50% after 4 h of dialysis. In another case, polysulfone–polyethylene glycol block-copolymer membranes achieved a lysozyme clearance of 46.4 ± 2.2 mL min^−1^, while BSA loss remained low at 8.7 ± 0.6 mL min^−1^ [16]. Beyond these conventional hydrophilic modifiers, recent progress on two-dimensional nanomaterials, such as MXenes and graphene oxide, has shown that nanoscale functional fillers can regulate interfacial interactions and mass-transport behavior in polymer hybrid membranes, thereby improving water transport, selectivity, and antifouling performance [17]. Nevertheless, many of these strategies primarily regulate phase-separation kinetics and surface hydrophilicity, while providing only limited control over the fraction of effective working pores, pore connectivity, and transport-pathway tortuosity. Consequently, achieving a satisfactory balance between middle-molecule toxin removal and protein retention remains challenging [18,19].

Therefore, a structural regulation strategy is still needed to modulate pore formation during membrane fabrication, thereby improving the accessibility of effective transport channels and pore connectivity while reducing apparent mass-transfer resistance. Accordingly, SBA-15 was incorporated into the PES matrix as an inorganic pore-regulating additive. SBA-15 possesses ordered mesoporous channels, a relatively narrow pore-size distribution, a rigid inorganic framework, and abundant surface silanol groups, making it suitable as a pore-structure-regulating unit during nonsolvent-induced phase separation (NIPS) membrane formation. The ordered mesoporous architecture of SBA-15, together with its tunable pore size typically in the range of 2–10 nm, may assist pore formation and improve the accessibility of effective transport pathways within the PES membrane. This design matches the size gap between representative middle molecules and albumin, whose hydrodynamic sizes are about 3.5 and 7.2 nm, respectively [20,21,22]. In addition, SBA-15 may promote the formation of additional effective transport pathways by improving local membrane wettability and regulating pore formation and apparent pore interconnection during membrane formation [23,24,25]. These effects may increase the fraction of effective working pores, reduce transport-pathway tortuosity, and decrease the transmembrane transport resistance of middle molecules.

**Figure 1:**
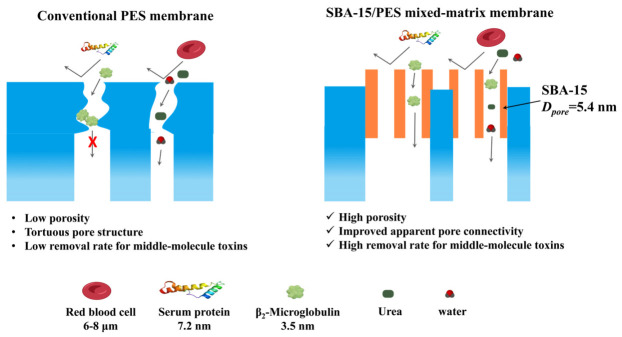
Schematic illustration of the proposed transport pathways and size-sieving behavior of pristine polyethersulfone (PES) and ordered mesoporous silica/polyethersulfone mixed-matrix membranes. The arrows indicate the proposed transport directions of water and solutes through the membrane pores, and the red cross indicates restricted transport through tortuous or constricted pores. The drawing is conceptual and not to scale, and it does not represent the exact pore geometry of the membranes.

In this study, SBA-15/PES mixed-matrix membranes with different SBA-15 loadings were prepared by NIPS. The analysis focused on how SBA-15 affected membrane morphology, pore structure, wettability, separation behavior, and protein-fouling resistance. PEG rejection tests and in vitro dialysis experiments were then used to link the changes in effective pore-size distribution and pore connectivity with middle-molecule transport. Therefore, this work examines whether SBA-15 can regulate the pore structure and apparent pore connectivity of PES membranes, thereby improving middle-molecule solute removal without weakening protein retention. The proposed transport pathways and size-sieving behavior are schematically illustrated in Figure 1.

## 2. Experimental Section

### 2.1. Materials

Polyethersulfone (PES, E6020P) and phosphate-buffered saline (PBS, 0.01 mol L^−1^, pH 7.4) were obtained from Source Leaf Biotechnology Co., Ltd. (Shanghai, China). Polyvinylpyrrolidone (PVP, K90) and N, N-dimethylacetamide (DMAc) were supplied by J&K Scientific Ltd. (Beijing, China) and Aladdin Chemistry Co., Ltd. (Shanghai, China), respectively. Tetraethyl orthosilicate (TEOS, 99%), Pluronic P123, hydrochloric acid, and absolute ethanol were commercially purchased and used for SBA-15 synthesis. Urea, lysozyme, bovine serum albumin (BSA), and polyethylene glycol standards (PEG, 2000–35,000 Da) were used in the separation and pore-size tests. L929 cells, cell culture reagents, fresh sterile sodium citrate-anticoagulated rabbit whole blood (10 mL/bottle; Guangzhou Hongquan Biotechnology Co., Ltd., Guangzhou, China), and coagulation assay kits were used for biocompatibility evaluation. Unless stated otherwise, all reagents were used without further purification.

### 2.2. Synthesis of SBA-15 Mesoporous Sieve

SBA-15 was synthesized following a reported hydrothermal route with minor adjustments [26]. Briefly, concentrated HCl, deionized water, P123, and TEOS were mixed at a molar ratio of P123:HCl:H_2_O:TEOS = 1:341:11,353:72. In a typical synthesis, 9.63 g of concentrated HCl and 52.49 g of deionized water were mixed at 40 °C, followed by the addition of 2.09 g of P123. After 4 h of stirring, 5.35 g of TEOS was added dropwise, and the suspension was kept under stirring at 40 °C for another 24 h. The mixture was then sealed in a 100 mL Teflon-lined autoclave and crystallized at 100 °C for 48 h. The product was recovered by centrifugation, washed with deionized water and ethanol, dried overnight at 80 °C, and calcined in air at 550 °C for 6 h.

### 2.3. Preparation of SBA-15/PES Mixed-Matrix Membranes

SBA-15/PES mixed-matrix membranes were fabricated by NIPS [27]. Before use, PES, PVP, and SBA-15 were dried at 80 °C for 12 h. The casting solutions were prepared according to the formulations in Table 1. Briefly, PVP and PES were first dissolved in DMAc at 60 °C, and SBA-15 was then added under stirring. The suspension was stirred for 24 h to ensure uniform dispersion, followed by vacuum degassing at 30 °C for 12 h. Before casting, the casting solution was equilibrated at 25 °C. The pristine PES membrane was prepared in the same way, but without SBA-15. Deionized water was used as the coagulation bath because it is a strong nonsolvent for the PES/DMAc/PVP casting system. The same coagulation bath was used for all membranes to maintain consistent NIPS conditions and to compare the effect of SBA-15 loading.

The casting solution was cast on a clean glass plate with a 300 μm casting gap at 10 cm s^−1^. The wet film was immediately immersed in a deionized water coagulation bath at 25 °C to complete phase separation. The membranes were then kept in deionized water for 24 h to remove residual DMAc and dried at 30 °C for 12 h.

### 2.4. Membrane Characterizations

Membrane morphology was examined by field-emission SEM (SU8010, Hitachi, Tokyo, Japan). Surface and cross-sectional samples were sputter-coated with gold before observation, and the accelerating voltage was 15 kV. Elemental mapping was performed with an EDS detector coupled to the SEM. The mesoporous channels of SBA-15 were observed by TEM (HT7700, Hitachi, Tokyo, Japan) at 200 kV. Low-angle XRD patterns were collected on an XRD-6100 diffractometer (Shimadzu, Kyoto, Japan) over 2θ = 0°–10° to verify the ordered mesostructure of SBA-15. N_2_ adsorption–desorption isotherms were measured using an ASAP 2460 analyzer (Micromeritics, Norcross, GA, USA). The BET method was used to calculate the specific surface area, and the BJH model was applied to obtain the pore-size distribution. FT-IR spectra of the membranes were recorded on a Nicolet iS20 spectrometer (Thermo Fisher Scientific, Waltham, MA, USA) in the range of 4000–400 cm^−1^ with a resolution of 4 cm^−1^.

### 2.5. Membrane Performance Test

(1) Membrane porosity was measured by a gravimetric method [28]. Each dry membrane sample with a known area was weighed as $m_{0}$ and then soaked in deionized water for 4 h. After soaking, the sample was taken out, and surface water was gently removed with filter paper before the wet mass was recorded as $m_{1}$ Porosity was calculated using Equation (1):

(1) $$\varepsilon =\frac{\left(m_{1}-m_{0}\right)}{\rho \times A\times l}\times 100\%$$

where *ε* denotes porosity (%), $m_{1}$ and $m_{0}$ are the wet and dry membrane masses, respectively, ρ is the density of water at 25 °C, and A and l are the membrane area and thickness, respectively.

(2) Membrane wettability was determined from static water contact angles [29]. For each test, a dry membrane was placed flat on the sample stage, and 5 μL of ultrapure water was dropped onto its surface. The contact angle was recorded after droplet deposition. Five randomly selected points were measured for each membrane, and the average value was used.

(3) Pure water permeability (PWP) was tested with a laboratory-built filtration setup [30]. A wet circular membrane with a diameter of 37 mm was mounted in the cell, giving an effective area of 10.75 cm^2^. The membrane was first compacted with pure water at 1 bar for 1 h. After the flux reached a steady state, the permeate volume was recorded at 20 min intervals. PWP was calculated using Equation (2):

(2) $$PWP=\frac{∆V}{S\times ∆t\times ∆P}$$

where ∆V is the collected permeate volume, S is the effective membrane area, ∆t is the filtration time, and ∆P is the applied transmembrane pressure. *PWP* is reported in L m^−2^ h^−1^ bar^−1^.

(4) The molecular weight cut-off (MWCO) and effective pore-size distribution were determined from PEG rejection tests [31]. A dead-end filtration cell equipped with magnetic stirring at 300 rpm was used for the measurements. A wet membrane disk with a diameter of 37 mm was placed in the cell, corresponding to an effective area of 10.75 cm^2^. The tests were performed at 25 °C. Before PEG filtration, the membrane was compacted with ultrapure water at 1 bar for 1 h. After a stable water flux was reached, PEG solutions with molecular weights of 2000, 4000, 6000, 8000, 10,000, 15,000, 20,000, and 35,000 Da were tested in sequence. The concentration of each PEG solution was 200 mg L^−1^, and a fresh solution was used for each run without recycling. PEG concentrations in the feed and permeate were measured by the iodine–potassium iodide colorimetric method. Each test was repeated three times. PEG rejection was calculated using Equation (3):

(3) $$R=\left(1-\frac{C_{P}}{C_{f}}\right)\times 100\%$$

where $C_{p}$ and $C_{f}$ are the PEG concentrations in the permeate and feed, respectively.

The Stokes radius (rp) of PEG was calculated using Equation (4):

(4) $$rp=6.73\times 10^{-3}\times M^{0.557}$$

where *M* is the molecular weight of PEG (g mol^−1^).

The effective pore-size distribution of the membranes was fitted using a log-normal distribution model, as shown in Equation (5):

(5) $$\frac{dR(d_{p})}{d(d_{p})}=\frac{1}{d_{p}\ln\sigma_{p}\sqrt{2\pi }}\exp\left[-\frac{(\ln d_{p}-\ln\mu_{p}{)}^{2}}{2(\ln\sigma_{p}{)}^{2}}\right]$$

where $\mu_{p}$ represents the mean effective pore size, and $\sigma_{p}$ is the geometric standard deviation of the pore-size distribution.

### 2.6. In Vitro Dialysis Simulation

The separation performance under dialysis conditions was tested with a self-assembled in vitro dialysis system. Urea, lysozyme, and BSA were selected as model solutes representing small-molecule toxins, middle-molecule toxins, and albumin, respectively [32]. Lysozyme in PBS was used as a proxy for middle molecules to isolate membrane effects from complex plasma interactions. The feed solution was PBS (pH 7.4) containing urea (1 g L^−1^), lysozyme (0.04 g L^−1^), and BSA (1 g L^−1^), and the dialysate was PBS at the same pH. During the test, the feed and dialysate flowed in opposite directions at 200 and 500 mL min^−1^, respectively. The experiment was conducted at 37 °C for 4 h. At 1 h intervals, 5 mL of solution was sampled from both sides. Urea was measured by the p-dimethylaminobenzaldehyde colorimetric method, and the concentrations of urea, lysozyme, and BSA were obtained from UV–Vis calibration curves. Each test was performed in triplicate. Solute reduction and BSA retention were calculated using Equations (6) and (7):

(6) $$C=\left(1-\frac{C_{f,t}}{C_{f,0}}\right)\times 100\%$$

(7) $$R_{e}=\left(1-\frac{C_{d,t}}{C_{f,t}}\right)\times 100\%$$

where $C_{f,0}$ and $C_{f,t}$ are the solute concentrations in the feed solution before dialysis and at time t, respectively, $C_{d,t}$ and $C_{f,t}$ are the BSA concentrations in the dialysate and feed solution at time t, respectively.

### 2.7. Antifouling Performance Evaluation

(1) Protein adsorption was quantified using BSA as the model protein [33]. Membrane pieces of 1.0 cm × 1.0 cm were immersed in PBS (pH 7.4) containing 5 mg mL^−1^ BSA and incubated at 37 °C for 4 h. After incubation, the membranes were rinsed with PBS to remove weakly attached protein. The adsorbed BSA was then desorbed by soaking the membranes in 2 wt.% SDS solution at room temperature for 2 h. The protein concentration in the SDS solution was measured by UV–Vis spectrophotometry, and the adsorption amount was normalized to the membrane area using Equation (8):

(8) $$Q=\frac{C\times V}{A}$$

where Q is the protein adsorption amount per unit area, C is the protein concentration in the SDS desorption solution, V is the volume of the desorption solution, and A is the membrane area.

(2) Membrane antifouling behavior was examined by a BSA filtration–cleaning cycle. Before fouling, the membrane was compacted with pure water at 1 bar for 1 h, and the stabilized pure water permeability was recorded as $J_{w1}$. The feed was then changed to a BSA solution, and the average filtration flux was denoted as $J_{p}$. After BSA filtration, the membrane was rinsed with deionized water for 30 min, and the pure water permeability after cleaning was measured as $J_{w2}$ [34]. The flux recovery ratio (FRR), reversible fouling ratio ($R_{r}$), irreversible fouling ratio ($R_{ir}$), and total fouling ratio ($R_{t}$) were calculated using Equations (9)–(12):

(9) $$FRR=\frac{J_{W2}}{J_{W1}}\times 100\%$$

(10) $$Rr=\frac{J_{W2}-J_{p}}{J_{w1}}\times 100\%$$

(11) $$Rir=\left(\frac{J_{w1}-J_{w2}}{J_{w1}}\right)\times 100\%$$

(12) Rt=Rr+Rir

where $J_{w1}$ is the initial pure water permeability, $J_{w2}$ is the pure water permeability after cleaning, and $J_{p}$ is the average flux during BSA filtration.

Unless otherwise stated, all quantitative experiments were performed at least three times, and the results are presented as mean ± standard deviation.

## 3. Results and Discussion

### 3.1. Structural Characteristics of SBA-15 Mesoporous Molecular Sieve

**Figure 2:**
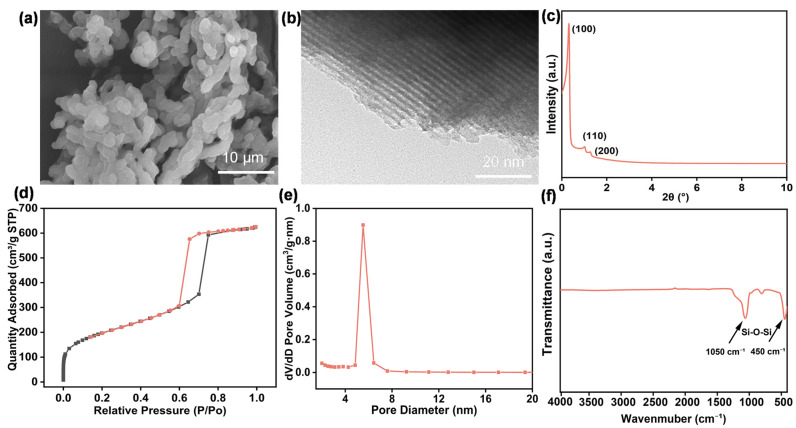
Morphological and structural characterization of SBA-15. (**a**) SEM image; (**b**) TEM image; (**c**) XRD pattern; (**d**) N_2_ adsorption–desorption isotherm, the black and red curves represent the adsorption and desorption branches, respectively; (**e**) BJH pore-size distribution, (**f**) FT-IR spectrum.

SEM, TEM, XRD, FT-IR, and N_2_ adsorption–desorption measurements were performed to characterize the morphology and mesoporous structure of SBA-15. As shown in Figure 2a, the obtained SBA-15 appears as short rod-like particles with loose aggregation. The TEM image in Figure 2b shows parallel channels along the particle axis, confirming the ordered mesoporous arrangement. In the low-angle XRD pattern (Figure 2c), three reflections assigned to the (100), (110), and (200) planes are observed, which is consistent with the two-dimensional hexagonal structure of SBA-15 [35]. The FT-IR spectrum (Figure 2f) displays bands near 1050 and 450 cm^−1^, corresponding to Si–O–Si asymmetric stretching and framework bending vibrations, respectively [36]. The N_2_ adsorption–desorption curve (Figure 2d) exhibits a type IV isotherm with a clear hysteresis loop. The BJH result (Figure 2e) gives a main pore-size peak at about 5.4 nm, indicating that SBA-15 has an ordered mesoporous structure with a narrow pore-size distribution [37,38].

### 3.2. Morphological and Structural Characteristics of SBA-15/PES Mixed-Matrix Membranes

**Figure 3:**
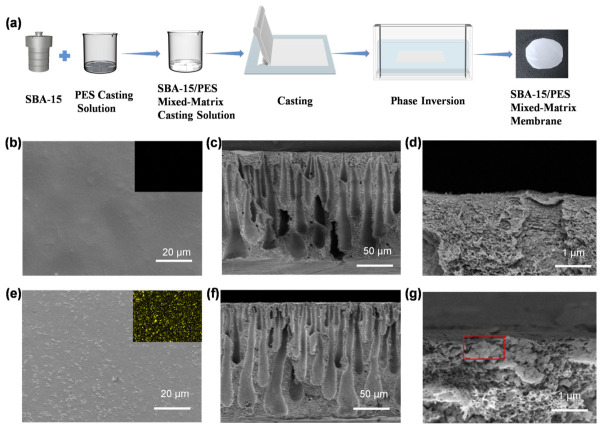
NIPS fabrication and SEM morphology of SBA-15/PES mixed-matrix membranes: (**a**) Schematic of the membrane preparation process; (**b**–**d**) surface, cross-sectional, and enlarged cross-sectional SEM images of the pristine PES membrane (M0); and (**e**–**g**) corresponding SEM images of the 7 wt.% SBA-15/PES membrane (M4). The red box in panel (**g**) highlights the locally exposed filler-rich region near the membrane surface.

To examine how SBA-15 changed the membrane structure, M0 and M4 were compared in the main text because they represent the pristine PES membrane and the membrane with the best overall performance, respectively (Figure 3). SEM images of the remaining samples are provided in Figure S1 [39,40,41,42]. As illustrated in Figure 3a, the membranes were prepared by NIPS. SBA-15 was dispersed in the PES dope solution before casting. After the dope solution was cast onto the substrate, the wet film was transferred into a nonsolvent bath, where solvent–nonsolvent exchange drove phase separation and membrane formation.

Surface SEM images show that M0 has a relatively smooth surface (Figure 3b), whereas M4 exhibits pronounced granular surface undulations (Figure 3e). Combined with Si elemental EDS mapping, SBA-15 is observed to be distributed within the membrane surface region, with local partial exposure of the filler (Figure 3g). Cross-sectional SEM images reveal that both membranes have a typical asymmetric structure, with a dense, selective layer on top and a finger-like porous support layer beneath (Figure 3c,f). Compared with M0, M4 displays more continuous finger-like pores with a higher degree of vertical penetration, suggesting that the incorporation of SBA-15 altered the phase-separation behavior during NIPS and promoted the formation of a more open supporting structure [43]. Higher-magnification cross-sectional images further show locally exposed filler domains near the surface of M4. These domains may improve local wettability near the selective layer and participate in pore-structure regulation, which may help form additional effective transport pathways in the PES membrane and reduce apparent transmembrane mass-transfer resistance.

In addition, SBA-15 incorporation may affect the flow behavior of the casting solution and the phase-separation process. Under the same testing conditions, the apparent viscosities of the representative casting solutions M0, M4, and M5 were 1814, 2944, and 3387 mPa·s, respectively, indicating that SBA-15 increased the apparent viscosity of the casting solution. This increase may be attributed to the higher inorganic filler content, lower relative DMAc content, and possible interactions between SBA-15 and the PES/PVP chains or solvent molecules. A moderate increase in apparent viscosity may help regulate the NIPS process, whereas excessive SBA-15 loading may hinder filler dispersion and solvent–nonsolvent exchange, leading to reduced pore-structure uniformity in M5. This is consistent with its decreased pure water permeability and dialysis performance.

XRD and FT-IR analyses were further performed to verify the incorporation of SBA-15 into the PES membranes and the retention of its ordered structure after the NIPS process. The results are presented in Figures S2 and S3. For M1–M5, the low-angle XRD peaks of SBA-15 are still visible, showing that the ordered mesoporous structure was largely retained after membrane formation (Figure S2). The FT-IR spectra also show the main absorption bands of PES together with Si–O–Si signals from SBA-15, supporting the successful introduction of SBA-15 into the PES matrix (Figure S3).

### 3.3. Pure Water Permeability, Porosity, and Hydrophilicity

**Figure 4:**
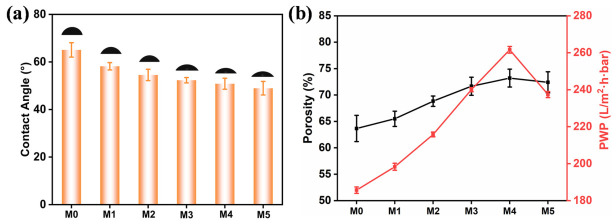
Hydrophilicity and transport-related properties of SBA-15/PES mixed-matrix membranes with different SBA-15 loadings: (**a**) water contact angle; (**b**) porosity and PWP. In panel (**a**), the black semicircles represent the representative water droplet profiles used for contact-angle measurement. In panel (**b**), the black and red curves represent porosity and PWP, respectively. Data are presented as mean ± SD (*n* = 3).

The changes in hydrophilicity, porosity, and pure water permeability (PWP) as a function of SBA-15 loading are summarized in Figure 4. As shown in Figure 4a, the water contact angle decreased from 65.1° for M0 to 49.0° for M4, indicating that SBA-15 improved the membrane surface hydrophilicity. This improvement is related to the silanol groups on SBA-15 [44]. The porosity and PWP followed a similar trend at moderate filler loadings. When the SBA-15 loading increased from 0 to 7 wt.%, porosity rose from 63.7% to 73.2%, and PWP increased from 185.6 to 261.5 L m^−2^ h^−1^ bar^−1^ (Figure 4b). In agreement with the SEM observations, these changes indicate that SBA-15 helped form more continuous finger-like pores and improved internal pore connectivity [45]. The ordered mesopores of SBA-15 may also offer additional transport routes for water, lowering the resistance across the membrane. However, at 9 wt.% SBA-15, porosity and PWP decreased to 72.3% and 237.3 L m^−2^ h^−1^ bar^−1^, respectively. This decline may be associated with partial filler aggregation or pore blockage. In addition, excessive SBA-15 loading may alter the rheological behavior of the casting solution, for example, by increasing apparent viscosity and reducing particle dispersion, thereby disturbing uniform pore formation during phase separation. Therefore, SBA-15 improves membrane hydrophilicity and transport only within an appropriate loading range, with M4 showing the best overall balance.

To further quantify the effect of SBA-15 on the apparent transport-related structure of the membranes, the apparent tortuosity and porosity–tortuosity ratio were calculated, as shown in Figure S4. The apparent tortuosity decreased from 5.00 for M0 to 2.06 for M4, whereas the porosity–tortuosity ratio increased from 12.71% to 35.47%. When the SBA-15 loading was further increased to 9 wt.% (M5), the apparent tortuosity increased to 2.50, and the porosity–tortuosity ratio decreased to 28.89%. These results indicate that an appropriate SBA-15 loading can reduce the apparent tortuosity and improve the effective transport capacity of the membrane, whereas excessive SBA-15 loading may diminish this benefit. Mechanical testing further showed that the maximum tensile stress generally increased, whereas the elongation at break decreased with increasing SBA-15 loading (Figure S5), indicating enhanced rigidity and reduced flexibility of the mixed-matrix membranes.

### 3.4. Effective Pore-Size Distribution

**Figure 5:**
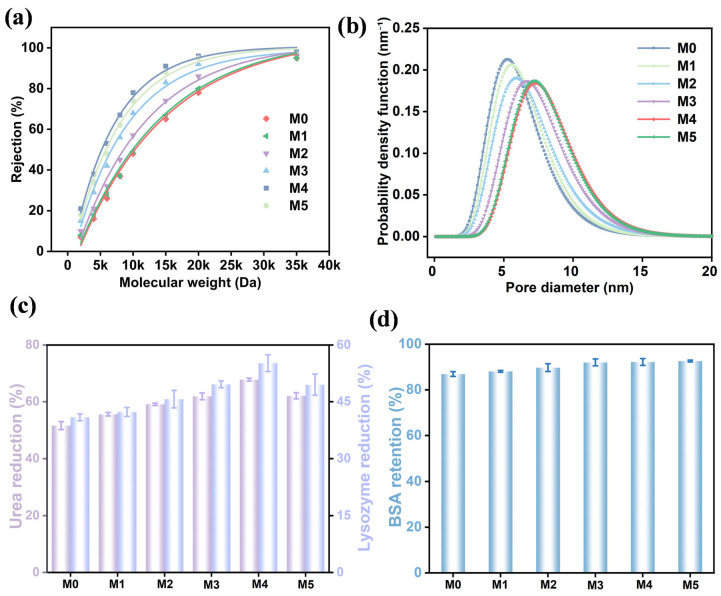
Pore-size analysis and separation performance of SBA-15/PES mixed-matrix membranes with different SBA-15 loadings: (**a**) PEG rejection curves; (**b**) effective pore-size distribution; (**c**) urea and lysozyme reduction; and (**d**) BSA retention. In panel (**c**), the purple and blue bars represent urea and lysozyme reduction, respectively. Data are presented as mean ± SD (*n* = 3).

PEG rejection tests were used to evaluate the effective sieving pores of the membranes, and the results are presented in Figure 5a,b [46]. From M0 to M4, the PEG rejection curves shifted toward lower molecular weights, and the rejection at the same molecular weight increased. This result indicates that the effective sieving ability of the membrane was enhanced after SBA-15 incorporation. The calculated pore-size distribution showed a similar tendency. As the SBA-15 loading increased to 7 wt.%, the main pore-size peak moved gradually toward smaller values, suggesting that SBA-15 helped regulate the effective pores in the selective surface layer.

Together with the SEM and PWP results, these findings suggest that an appropriate SBA-15 loading improved apparent internal pore connectivity and regulated the effective sieving pores in the selective layer. However, when the SBA-15 loading was increased to 9 wt.%, the PEG rejection curve shifted slightly toward higher molecular weights, and the rejection at a given molecular weight decreased. The pore-size distribution peak also moved slightly toward larger pores. This reverse trend suggests that excessive SBA-15 weakened the pore-regulating effect, possibly because filler aggregation or disturbed phase separation made the surface sieving pores less uniform. Figure S1 shows a less uniform Si distribution and local filler-rich regions in the M5 membrane, supporting this interpretation. These observations are consistent with the decreased porosity and pure water permeability of M5, suggesting that excessive SBA-15 loading may partially restrict effective transport pathways.

### 3.5. Dialysis Separation Performance

The dialysis separation results are summarized in Figure 5c,d. Urea and lysozyme were selected as model small-molecule and middle-molecule solutes, respectively, while BSA was used to evaluate protein retention. From M0 to M4, urea reduction increased from 51.6% to 65.5%, and lysozyme reduction increased from 40.9% to 55.2%. This improvement was consistent with the trends in PWP and pore connectivity, suggesting that a suitable amount of SBA-15 reduced the transport resistance inside the membrane. The SEM and PEG rejection results further support this conclusion. The incorporation of SBA-15 may help form additional effective transport pathways in the PES membrane by regulating pore formation and improving apparent pore connectivity. Consequently, the apparent transport resistance for urea and lysozyme was reduced under the tested in vitro conditions. The increase was larger for lysozyme, indicating that middle-molecule transport benefits more from pore-structure regulation than small-molecule transport.

When the SBA-15 loading reached 9 wt.%, urea and lysozyme reductions decreased to 62.1% and 50.2%, respectively. This decrease agrees with the lower PWP and porosity observed at high filler loading. It suggests that excessive SBA-15 no longer improves transport, but may partly block the pore network or disturb phase separation because of local filler aggregation. As a result, the transport resistance for urea and lysozyme increased. In contrast, BSA retention remained high for all membranes and showed a slight increase with SBA-15 loading (Figure 5d). This means that SBA-15 did not weaken the membrane barrier against large proteins within the tested loading range. The high BSA retention can be explained from two aspects. First, the improved surface hydrophilicity favors the formation of a hydration layer at the membrane–solution interface, which helps reduce BSA adhesion and transport across the membrane [19,36]. Second, SBA-15 possesses ordered mesoporous channels and abundant surface silanol groups, which may regulate pore formation, improve local hydrophilicity, and enhance apparent pore connectivity in the PES membrane. These effects may help form additional effective transport pathways for small-molecule and model middle-molecule solutes. Meanwhile, the selective layer still maintains a sufficient size-sieving barrier against large proteins such as BSA, thereby preserving protein retention [47,48,49].

To further assess albumin retention under a higher-albumin-loading condition, an additional 40 g L^−1^ BSA dialysis test was performed for M0 and M4. As shown in Figure S6 and Table S1, M4 showed a lower BSA sieving coefficient and cumulative albumin loss than M0 under both 1 and 40 g L^−1^ BSA conditions, indicating better albumin retention under the tested high-BSA-loading model condition.

Overall, incorporating an appropriate amount of SBA-15 can simultaneously improve solute reduction and protein retention. Under identical benchtop dialysis conditions, M4 showed the highest percentage reductions in feed urea and lysozyme while maintaining >90% BSA retention. Because the values in Figure 5c represent percentage reductions measured under identical benchtop dialysis conditions rather than area-normalized clearances or KoA, they are used here mainly for relative comparison among the membranes. These results suggest that SBA-15 can serve as an effective structural regulator to optimize the membrane mass-transfer channel network, thereby providing a favorable structural basis for the design of hemodialysis membranes that simultaneously enhance middle-molecule toxin removal and protein retention.

To provide a preliminary assessment of possible SBA-15 leaching during dialysis, the Si concentration in the solution after the 4 h in vitro dialysis test was measured, and the values for the PBS blank, M0, and M4 groups were 0.08, 0.11, and 0.45 mg L^−1^, respectively, suggesting limited Si release from the SBA-15/PES membrane under the tested in vitro conditions.

The optimized membrane was further benchmarked against reported dialysis membranes, as shown in Table 2. The comparison was based on urea reduction, lysozyme reduction, and BSA retention. Because the testing systems, solute compositions, and operating conditions used in different studies are not fully identical, this comparison should be regarded mainly as a reference-based performance comparison. The SBA-15/PES membrane prepared in this work showed a urea reduction of 65.5% and a lysozyme reduction of 55.2%, while keeping BSA retention at 92.4%. Compared with the reported membranes, this membrane provided a favorable combination of middle-molecule reduction and protein retention under the tested in vitro conditions. These results suggest that SBA-15 helps tune the effective pore structure and mass-transfer pathway of PES membranes.

### 3.6. Antifouling Performance

Protein antifouling performance was further analyzed for SBA-15/PES membranes containing 1–7 wt.% SBA-15. These samples were compared in terms of BSA adsorption, flux change during dynamic filtration, flux recovery, and fouling resistance. The 9 wt.% membrane was not emphasized in this section because, as discussed above, its porosity, PWP, and dialysis separation performance decreased, probably due to filler aggregation and partial restriction of effective transport pathways.

**Figure 6:**
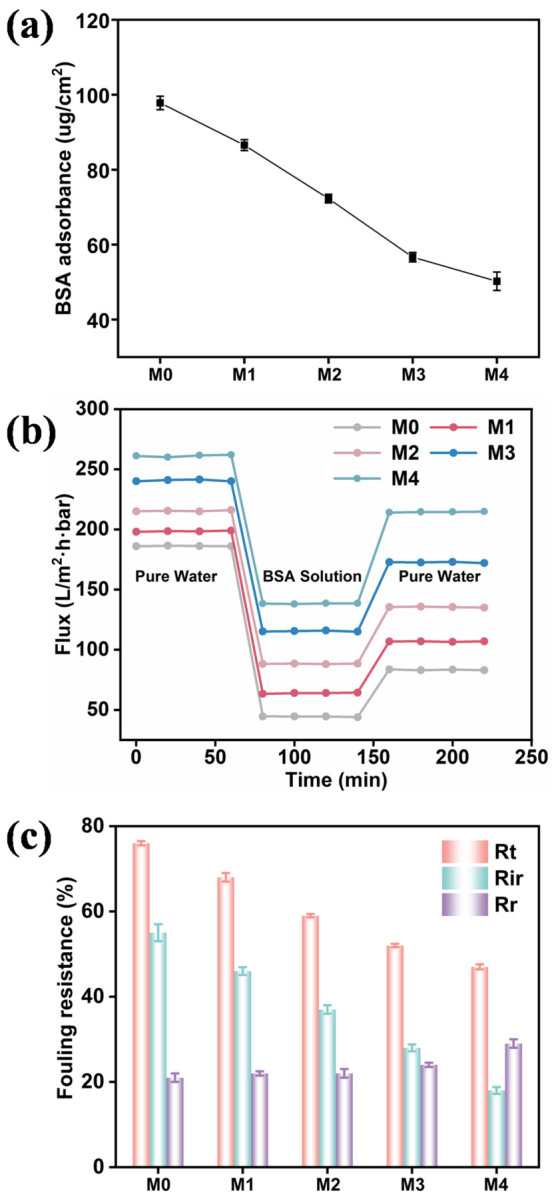
Antifouling behavior of SBA-15/PES mixed-matrix membranes with different SBA-15 loadings: (**a**) BSA adsorption; (**b**) flux change during the water/BSA/water filtration cycle; and (**c**) fouling resistance distribution. Data are presented as mean ± SD (*n* = 3).

The static adsorption results show that BSA adsorption decreased gradually as the SBA-15 loading increased from 1 to 7 wt.% (Figure 6a). Compared with pristine PES, the SBA-15/PES membranes adsorbed less BSA, indicating a lower tendency toward protein attachment. This behavior agrees with the contact-angle results. The improved surface hydrophilicity can help form a hydration layer on the membrane surface, which weakens direct contact between BSA and the membrane and therefore reduces protein adsorption [55].

The dynamic filtration results further confirm the antifouling effect of SBA-15 (Figure 6b,c). During BSA filtration, all membranes showed a flux decline, but the decline was slower for the SBA-15/PES membranes than for pristine PES. With increasing SBA-15 loading, the flux during fouling remained higher, and the recovered flux after cleaning also improved [56,57,58]. The resistance analysis shows that total fouling resistance ($R_{t}$) decreased with SBA-15 loading, mainly because irreversible fouling resistance ($R_{ir}$) was strongly reduced. In comparison, reversible fouling resistance ($R_{r}$) changed only slightly and showed a small increase for M4. These results indicate that SBA-15 reduces protein adsorption and pore blocking, especially the irreversible part of fouling. As a result, the fouling layer becomes easier to remove during cleaning, leading to better flux stability and recovery under BSA-fouling conditions.

Additional coagulation, hemolysis, and cytotoxicity tests were performed to evaluate the hemocompatibility and cytocompatibility of the membranes. Compared with pristine PES, the SBA-15/PES mixed-matrix membranes showed only slight prolongation of APTT, PT, and TT, and all values remained within the normal physiological range (Figure S7) [59,60]. The hemolysis ratios of all membranes were below 0.1%, far lower than the commonly used 5% threshold for blood-contacting materials (Figure S8), and the viability of L929 cells was higher than 85% (Figure S9) [61]. These results indicate that SBA-15 incorporation did not cause obvious coagulation abnormalities, red blood cell damage, or cytotoxicity under the in vitro test conditions. Therefore, the SBA-15/PES mixed-matrix membranes showed acceptable preliminary hemocompatibility and cytocompatibility for hemodialysis-related membrane evaluation.

## 4. Conclusions

In this work, SBA-15/PES mixed-matrix membranes were prepared by NIPS, and the role of SBA-15 in regulating membrane structure and dialysis separation performance was investigated. The incorporation of SBA-15 may help form additional effective transport pathways in the PES membrane by regulating pore formation, increasing surface hydrophilicity, and improving apparent pore connectivity. These structural changes were associated with reduced apparent mass-transfer resistance and improved solute transport. Among the prepared membranes, M4 with 7 wt.% SBA-15 showed the best overall performance, with a PWP of 261.5 L m^−2^ h^−1^ bar^−1^, urea and lysozyme reduction of 65.5% and 55.2%, respectively, and BSA retention of 92.4%. M4 also showed reduced protein adsorption and lower irreversible fouling. In addition, coagulation, hemolysis, and cytotoxicity results indicated acceptable preliminary hemocompatibility and cytocompatibility under the in vitro evaluation conditions. Overall, under buffered model conditions, SBA-15 improved model middle-molecule transport while maintaining albumin retention; validation with β_2_-microglobulin in plasma is warranted. This study provides a feasible route for designing dialysis-related membranes with improved transport and antifouling performance.

## Figures and Tables

**Table 1:** Composition of casting solution.

Sample	PES (wt.%)	PVP (wt.%)	DMAc (wt.%)	SBA-15 (wt.%)
M0	15	3	82	0
M1	15	3	81	1
M2	15	3	79	3
M3	15	3	77	5
M4	15	3	75	7
M5	15	3	73	9

**Table 2:** Comparison of separation performance of different dialysis membranes.

Membrane	Urea Reduction	Lysozyme Reduction	BSA Retention	Reference
DA-g-GOCOOH/PLA	65%	24.5%	95.6%	[50]
f-MWCNT/PVP90/PES	55%	27.9%	90%	[51]
f-MWCNT/S-PES	60%	33%	95%	[52]
E-TA/G/PES	54.29%	37.50%	92%	[53]
L-PEtOX100/TA/PES	63%	50%	94%	[54]
SBA-15/PES	65.5%	55.2%	92.4%	This work

## Data Availability

The original contributions presented in this study are included in the article and Supplementary Materials. Further inquiries can be directed to the corresponding authors.

## Supplementary Materials

The following supporting information can be downloaded at: https://www.mdpi.com/article/doi/s1, Figure S1: Morphological characterization of SBA-15/PES mixed-matrix membranes with different SBA-15 loadings; Figure S2: X-ray diffraction (XRD) patterns of SBA-15 and SBA-15/PES mixed-matrix membranes with different SBA-15 loadings; Figure S3: FT-IR spectra of the pristine PES membrane (M0), SBA-15, and SBA-15/PES mixed-matrix membranes with different SBA-15 loadings; Figure S4: Calculated apparent tortuosity and porosity–tortuosity ratio of SBA-15/PES mixed-matrix membranes with different SBA-15 loadings; Figure S5: Tensile stress–strain curves of SBA-15/PES mixed-matrix membranes with different SBA-15 loadings; Figure S6: Dialysis performance and albumin retention of M0 and M4 under different BSA concentrations; Table S1: BSA sieving coefficient and cumulative albumin loss of M0 and M4 under 1 and 40 g/L BSA feed conditions; Figure S7: Coagulation times of plasma after contact with different membrane samples; Figure S8: Hemolysis assay results of the membrane samples; Figure S9: Cytocompatibility evaluation of the membrane samples.
