# Supplementary material for: Fabrication of Ordered Mesoporous Silica/Polyethersulfone Mixed-Matrix Membranes for Improved Removal of Middle-Molecule Toxins Within Hemodialysis
Source: Membranes (Basel). 2026 Jul 21;16(7):250. doi: 10.3390/membranes16070250 (PMC13413485; doi:10.3390/membranes16070250)
Supplement: Supplementary file 1 [file membranes-16-00250-s001.zip › membranes-4373640-supplementary.pdf]

Supporting Information for

Fabrication of Ordered Mesoporous Silica/Polyethersulfone Mixed-Matrix Membranes for Improved Removal of Middle-Molecule Toxins Within Hemodialysis

Rongrong Ji [1], Peiyan Shi [1], Ting Dong [2], Wenjie Hou [2] and Kangjian Tang [1,*]

1 Innovation Center for Chemical Science, College of Chemistry, Chemical Engineering and Materials Science, Soochow University, Suzhou 215123, China; jirongronghb@163.com (R.J.); 9855428047@163.com (P.S.)

2 Department of Nephrology, The Fourth Affiliated Hospital of Soochow University, No. 9 Chongwen Road, Suzhou 215123, China; dongting@suda.edu.cn (T.D.); wjhou@suda.edu.cn (W.H.)

* Correspondence: kjtang@suda.edu.cn; Tel.: +86-0512-6588-0269

(a)

(b)

(c)

(d)

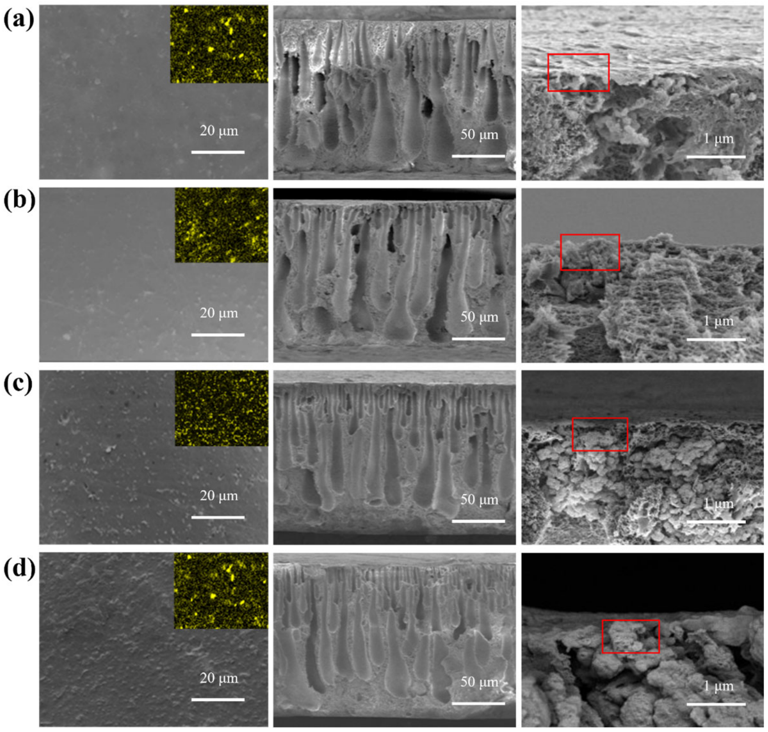

Figure S1. Morphological characterization of SBA-15/PES mixed-matrix membranes with different SBA-15 loadings: (a) M1, (b) M2, (c) M3, and (d) M5; for each membrane, the left, middle, and right panels correspond to the upper surface SEM image with Si elemental EDS mapping (inset), the entire cross-sectional SEM image, and the high-magnification cross-sectional SEM image, respectively.

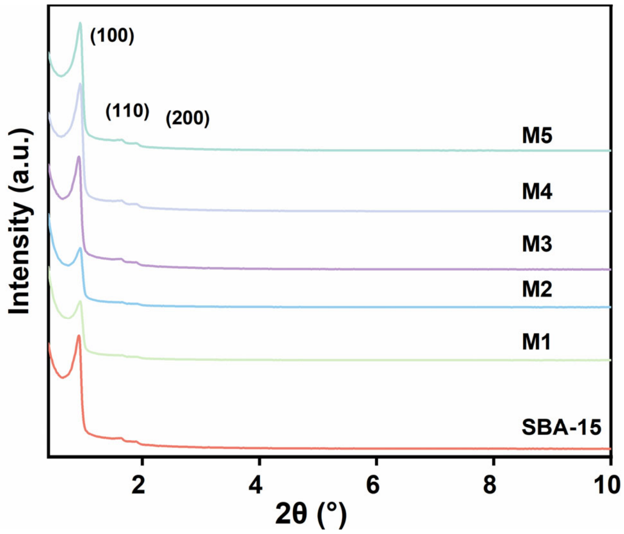

Figure S2. X-ray diffraction (XRD) patterns of SBA-15 and SBA-15/PES mixed-matrix membranes with different SBA-15 loadings (M1-M5).

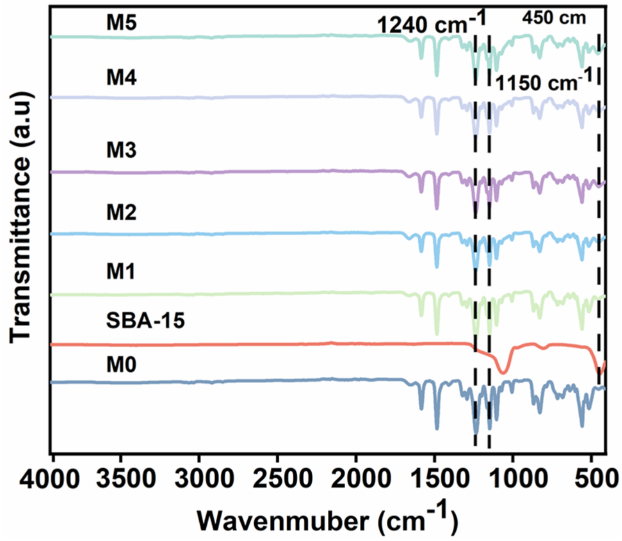

Figure S3. FT-IR spectra of the pristine PES membrane (M0), SBA-15, and SBA-15/PES mixed-matrix membranes with different SBA-15 loadings (M1-M5).

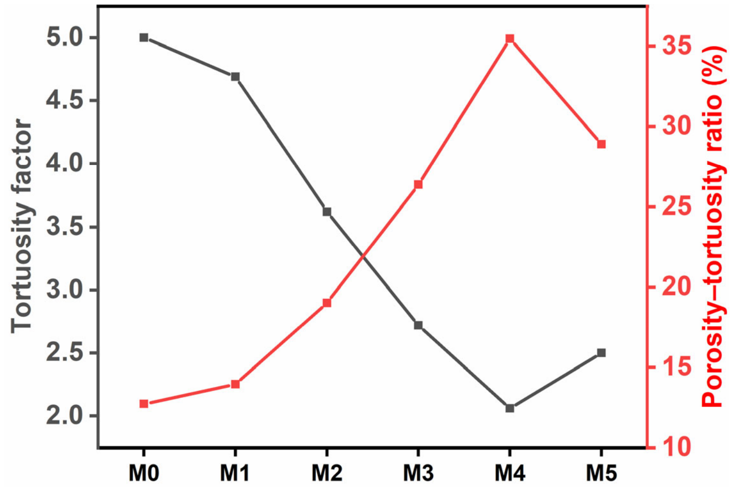

Figure S4. Calculated apparent tortuosity and porosity–tortuosity ratio of SBA-15/PES mixed-matrix membranes with different SBA-15 loadings.

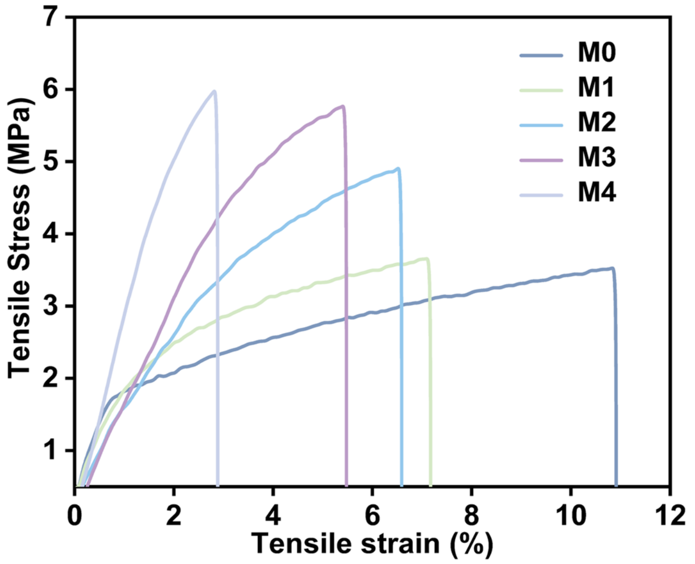

Figure S5. Tensile stress–strain curves of SBA-15/PES mixed-matrix membranes with different SBA-15 loadings.

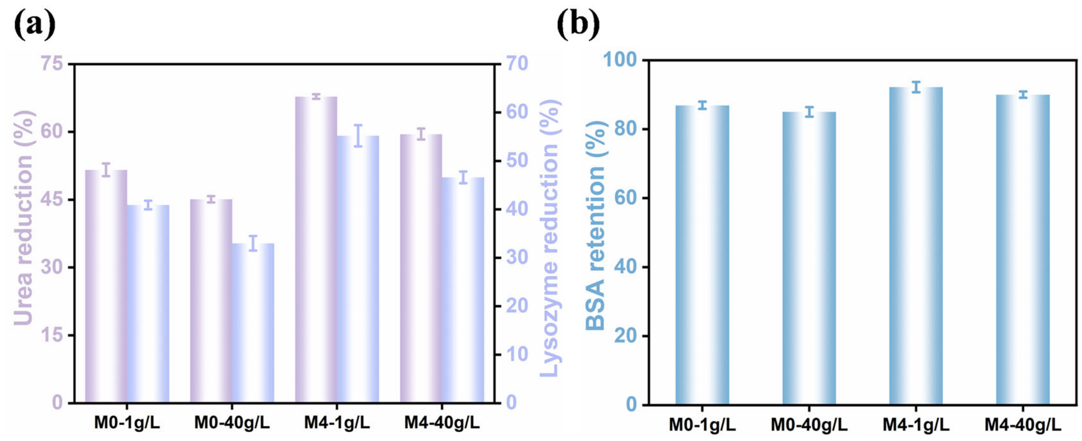

Figure S6. Dialysis performance and albumin retention of M0 and M4 under different BSA concentrations. (a) Urea and lysozyme reduction under 1 and 40 g/L BSA feed conditions. (b) BSA retention under 1 and 40 g/L BSA feed conditions. Data are presented as mean ± SD (n = 3).

Table S1. BSA sieving coefficient and cumulative albumin loss of M0 and M4 under 1 and 40 g/L BSA feed conditions.

Sample	BSA sieving coefficient	Cumulative albumin loss (mg)
M0-1g/L	0.131	0.085
M0-40g/L	0.15	4.0
M4-1g/L	0.076	0.067
M4-40g/L	0.096	3.2

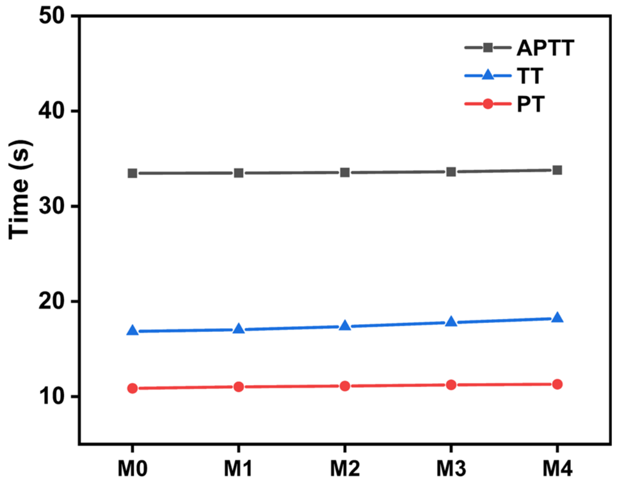

Figure S7. Coagulation times of plasma after contact with different membrane

samples: activated partial thromboplastin time (APTT), prothrombin time (PT), and thrombin time (TT).

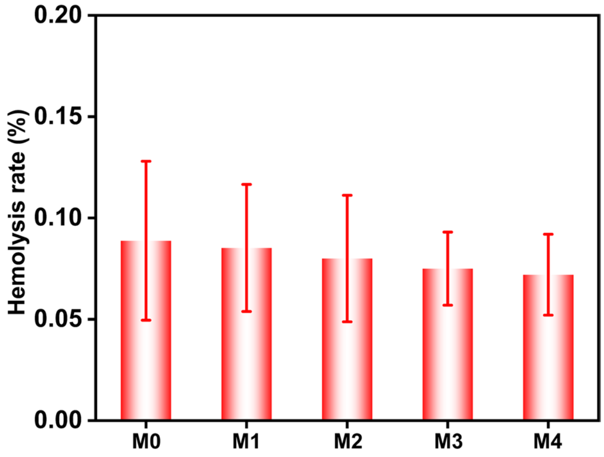

Figure S8. Hemolysis assay results of the membrane samples. Data are presented as mean ± SD (n = 3).

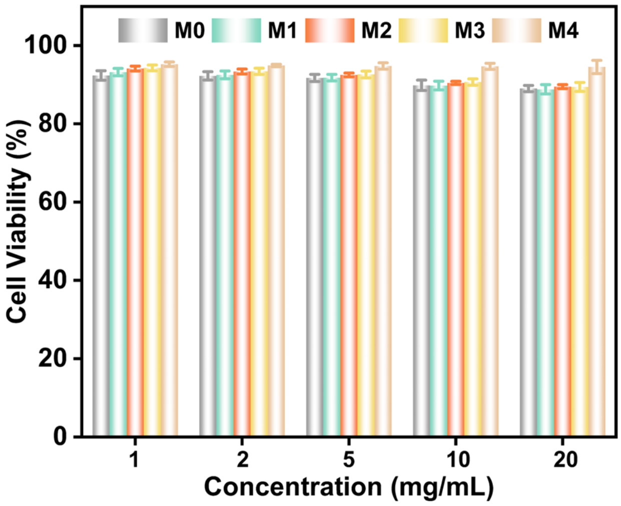

Figure S9. Cytocompatibility evaluation of the membrane samples: viability of L929 cells after exposure to membrane extracts at different concentrations. Data are presented as mean ± SD (n = 3).
